# Comparative proteomics analysis in different stages of urothelial bladder cancer for identification of potential biomarkers: highlighted role for antioxidant activity

**DOI:** 10.1186/s12014-023-09419-8

**Published:** 2023-07-27

**Authors:** Samira Tabaei, Mohammad Reza Haghshenas, Ali Ariafar, Kambiz Gilany, Allan Stensballe, Shirin Farjadian, Abbas Ghaderi

**Affiliations:** 1 Department of Immunology, School of Medicine, Shiraz University of Medical Sciences, Shiraz, Iran; 2 Shiraz Institute for Cancer Research, School of Medicine, Shiraz University of Medical Sciences, Shiraz, Iran; 3 Department of Urology, School of Medicine, Shiraz University of Medical Sciences, Shiraz, Iran; 4 Integrative Oncology Department, Breast Cancer Research Center, Motamed Cancer Institute, ACECR, Tehran, Iran; 5 Department of Health Science and Technology, Aalborg University, Gistrup, 9260 Denmark; 6 Clinical Cancer Research Center, Aalborg University hospital, Gistrup, 9260 Denmark

**Keywords:** Urothelial bladder cancer, Proteomics, Biomarker, Cellular responses

## Abstract

**Background:**

Non-muscle-invasive bladder cancer (NMIBC) has a high recurrence rate and muscle-invasive bladder cancer (MIBC) has unfavorable outcomes in urothelial bladder cancer (UBC) patients. Complex UBC-related protein biomarkers for outcome prediction may provide a more efficient management approach with an improved clinical outcome. The aim of this study is to recognize tumor-associated proteins, which are differentially expressed in different stages of UBC patients compared non-cancerous tissues.

**Methods:**

The proteome of tissue samples of 42 UBC patients (NMIBC n = 25 and MIBC n = 17) was subjected to two-dimensional electrophoresis (2-DE) combined with Liquid chromatography–mass spectrometry (LC–MS) system to identify differentially expressed proteins. The intensity of protein spots was quantified and compared with Prodigy SameSpots software. Functional, pathway, and interaction analyses of identified proteins were performed using geneontology (GO), PANTHER, Reactome, Gene MANIA, and STRING databases.

**Results:**

Twelve proteins identified by LC-MS showed differential expression (over 1.5-fold, p < 0.05) by LC-MS, including 9 up-regulated in NMIBC and 3 up-regulated in MIBC patients. Proteins involved in the detoxification of reactive oxygen species and cellular responses to oxidative stress showed the most significant changes in UBC patients. Additionally, the most potential functions related to these detected proteins were associated with peroxidase, oxidoreductase, and antioxidant activity.

**Conclusion:**

We identified several alterations in protein expression involved in canonical pathways which were correlated with the clinical outcomes suggested might be useful as promising biomarkers for early detection, monitoring, and prognosis of UBC.

**Supplementary Information:**

The online version contains supplementary material available at 10.1186/s12014-023-09419-8.

## Background

Urothelial bladder cancer (UBC) is the most prevalent cancer of the urinary system, with estimating incidence of 573,278 cases, globally [1]. Exposome factors like tobacco smoking and exposure to occupational toxins are the most principal risk factors that play a role in the occurrence of UBC [2]. According to molecular and pathological characteristics, UBC patients are divided into heterogeneous subgroups, including non-muscular invasive bladder cancer (NMIBC) (stages Ta and T1), and muscular invasive bladder cancer (MIBC) (stages T2 and above) [3, 4]. Approximately 80% of UBC patients initially are diagnosed with NMIBC whereas the remaining ~ 20% of the patients are diagnosed with MIBC which has a high progression rate and poor prognosis [5]. Despite, recent promising advances in clinical diagnostic methods, as well as, improvements in surgical and immune regional therapies, the high recurrence risk (between 50 and 70% of cases) of UBC remains challenging [6, 7].

Therefore, identification of tumor at early stages is paramount to prevent progression to metastasis of UBC patients [8, 9]. Considering the inconvenience, invasiveness, low sensitivity, and low specificity (particularly false negatives) of current diagnostic methods and lack of appropriate prognostic biomarkers for UBC a search for novel molecular biomarkers becomes more indispensable [6, 10].

Until now, various efforts have been made to characterize the potential biomarkers for UBC, among those, proteins are the most interesting biological targets [11, 12]. Protein biomarkers are critically important because any alterations in the protein profile significantly reflects the pathological conditions of tumors, which can be considered as effective targets for early cancer detection, regular screening, and immediate treatment solutions [13]. For investigating protein biomarkers, tumor tissue is exclusively important [14].

Thus far, several proteomics studies have proposed candidate proteins with differential expression biomarkers in UBC [15–17]. Strikingly, contradictory results due to the high heterogeneity of bladder tumors warrants further investigation particularly in clinical applications are needed.

This study aimed to recognize the proteome changes associated with UBC that could lead to a better insight into molecular mechanisms and pathophysiology of this cancer. Towards that end, we compared the proteome of tissue samples from NMIBC and MIBC patients using two-dimensional electrophoresis (2-DE) combined with a mass spectrometry (MS) technique. Through 2-DE, we identified several alterations in protein expression and found the canonical pathways were correlated with the clinical outcomes that may be useful as promising biomarkers for early detection, monitoring, and progression of UBC.

## Materials and methods

### Samples collection

In this study, samples were obtained from 42 newly diagnosed UBC patients who had undergone transurethral resection of bladder tumor (TURBT) and radical cystectomy. After, obtaining informed consent from each patient according to the Declaration of Helsinki and was approved by the local Ethics Committee of Shiraz University of Medical Sciences [IR.SUMS.REC.1400.369].

Samples from cancerous and adjacent non-cancerous tissues were collected from each patient from the surgery department of Namazi and Mother and Child university Hospitals (Shiraz, Iran) between 2021 and 2022. Fresh tumor tissues without any non-tumor tissue were washed three times with a cold PBS, snap frozen in liquid nitrogen, and stored at -80°C until the proteomics sample preparation and LC-MS analysis.

UBC patients with concurrent other malignant diseases, autoimmune diseases, previous history of TURBT chemotherapy, radiation therapy, or opium addiction as well as having a history of infectious diseases during a month before sampling were excluded from this study. All tumors were analyzed and characterized by an experienced pathologist. The tumor stage of UBC patients was determined according to the Union for International Cancer Control (UICC) (8th edition) and the 2016 World Health Organization criteria [18].

### Protein extraction

Aliquots of ~ 100 mg NMIBC and MIBC pooled tumor tissues were suspended in 1ml of urea lysis solution (7 M urea, 2 M thiourea, 40 mM dithiothreitol (DTT), 2% (w/v) 3-[(3-cholamidopropyl) dimethylammonio]-1-propanesulfonate (CHAPS), 2% (v/v) immobilized pH gradient (IPG) buffer (PH 3–10 NL), and one complete mini protease inhibitor cocktail tablet per liter. Afterward incubated at room temperature for 2 h with shaking to dissolve the tissue. Then, the lysates were centrifuged at 10,000 g for 20 min. The supernatant was collected and protein concentration was measured using Bio-Rad protein assay [19] with bovine serum albumin (BSA) as the standard. Following this, the protein lysates were stored at *-*80ºC until isoelectric focusing.

### Isoelectric focusing (IEF) and SDS-polyacrylamide gel electrophoresis (SDS-PAGE)

Extracted protein (proximally 500 µg) was added to thiourea rehydration buffer (7 M urea, 2 M thiourea 2 CHAPS (w/v), 1% bromophenol blue, and 2% (v/v) IPG buffer). The mixture was applied on 18-cm IPG gel strips (pH 3–10 nonlinear (NL)) (GE Healthcare Bio-Sciences AB, USA) and incubated at room temperature for 45 min. Then, actively rehydrated for 16 h at 50 V. Strips were then focused for 60000Vh at 20ºC within the BioRad Protean IEF Cell to separate proteins according to their isoelectric point (PI).

After IEF, the strips were equilibrated and reduced in the presence of equilibration buffer 6 M urea, 29.3% (v/v) glycerol, 2% (w/v) SDS, and 75 mM Tris-HCL containing 2% (w/v) DTT and then in the equilibration buffer containing 5% (w/v) iodoacetamide.

The equilibrated IPG strips were loaded onto the top of a 12% (w/v) SDS-PAGE and the proteins were separated according to their molecular weight (MW) using BioRad Protean IIxi. Then, the gels were visualized using a modified Coomassie brilliant blue G-250 (CBB) staining method.

### 2D-gel images analysis and protein mass spectrometry

The protein patterns in the stained 2D-gels were analyzed using the BioRad GS-800™ calibrated densitometer at a resolution of 300 dpi and recorded for further analysis. Spot detection and image matching were performed with SameSpots v5.1 software (Nonlinear Dynamics, Newcastle, UK). For image alignment protein gel maps of NMIBC group were considered as a reference for accurate analysis of differential expression. The labeled spots on each image were matched by SameSpots advanced algorithms based on their shape, size, and intensity.

Spots that were present in only one group or exhibited statistically significant (P < 0.05) and more than 1.5-fold changes in expression were selected for further identification using the timsTOF™ PRO2 LCMS system.

Excised protein spots were prepared for LC-MS based identification essentially according to [20]. Protein spots were destained by acetonitritrile (AcN, Sigma-Alrich)-100mM Ammoniumbicarbonate (Ambic, Sigma-Aldrich) incubation steps (10 min) at 0%, 50% and 100%AcN. At 100%AcN the gel plugs were dehydrated and remaining AcN evaporated by vacuum centrifugation. In-gel reduction and alkylation were performed at 10mM TCEP and 25mM chloroacetic acid (Sigma-Aldrich, CAA) for 30 min at 56 C followed washing step with dehydration by 50% and 100% AcN incubations. The shrinked gel-plugs were swelled in trypsin digestion buffer (0.5ug in 50mM Ambic) and incubated O/N at 37^ο^C. Peptides were extracted and peptide concentration measured by fluorescent quantitative peptide assays (Pierce). A total of 500ng peptide were loaded on Evotips according to manufacturer’s recommendations and analyzed by LC-MS using Evosep One (Evosep, Odense, Denmark) interfaced to a timsTOF PRO2 (Bruker, Bremen, Germany) using a 100SPD method and standard nESI-PASEF in positive ion mode. Protein identifications were accomplished by database searching against a human reference proteome database (UP000005640_9606; 20.460 proteins; NOV2022) using Maxquant (v. 2.0.3.0) with default settings and fixed modifications (carbamitometyl C) and variable (oxidizedM). Protein identifications were filtered and sorted according to number of peptides associated to the protein sequence, sequence coverage, theoretical pI and molecular weight in comparison to experimentally obtained pI for the analyzed gel-spot. Experimental raw and search outputs are available in the PRIDE repository (PXNNN).

### Protein interaction and pathway analysis

Gene ontology (GO) terms (http://geneontology.org) and PANTHER (protein analysis through evolutionary relationships) (http://pantherdb.org) web-based databases were conducted for categorizing identified proteins to their biological process, cellular component, and molecular function. Also, protein-protein interaction (PPI) networks of the selected proteins were performed using GeneMANIA (https://genemania.org), Reactome pathway, and STRING (Search Tool for the Retrieval of Interacting Genes/Proteins) v.11 − 5 database (https://string-db.org) with high confidence limit (< 0.70). The significant threshold of adjustment was considered to be P < 0.05.

### Statistical analysis

Prodigy SameSpots software package was performed to detect 2-DE spot differences between groups by the ANOVA and false discovery rate. Statistical analysis was conducted using the SPSS version 22.0 (SPSS Inc., Chicago, IL). P < 0.05 was considered statistically significant.

## Results

### Demographics and clinical characteristics of patients

To find biomarkers that can distinguish between different stages of UBC, tissue specimens from 42 patients (male, 31; female, 11; median age, 66.85 ± 9.61 years) were compared in three groups: NMIBC (Ta/T1, n = 25), MIBC (T2/T3/T4, n = 17), and adjacent non-cancerous (n = 10) group. The clinicopathological characteristics of UBC patients are summarized in Table 1.

**Table 1:** The clinicopathological characteristics of 42 urothelial bladder cancer patients

Clinicopathological characteristics	NMIBC (%) n = 25	MIBC (%) n = 17	p-value	Noncancerous (%) n = 10
**Gender**				
Male	18 (72)	13 (76.47)	0.74	7 (70)
Female	7 (28)	4 (23.52)		3 (30)
Age (mean ± SD)	66.48 ± 9.29	66.76 ± 11.62		66.10 ± 7.03
**Histological grade**				
Low grade	13 (52)	1 (5.88)	0.002*	-
High grade	12 (48)	16 (94.11)		-
**Tumor size (cm)**				
≦ 3	15 (60)	12 (70.58)	0.53	-
> 3	10 (40)	5 (29.41)		-
**Pathological T stage**				
	Ta/T1	T2/T3/T4		-

NMIBC, Non-muscle-invasive bladder cancer; MIBC, muscle-invasive bladder cancer; * p > 0.05

### Tissue proteome data

**Fig. 1:**
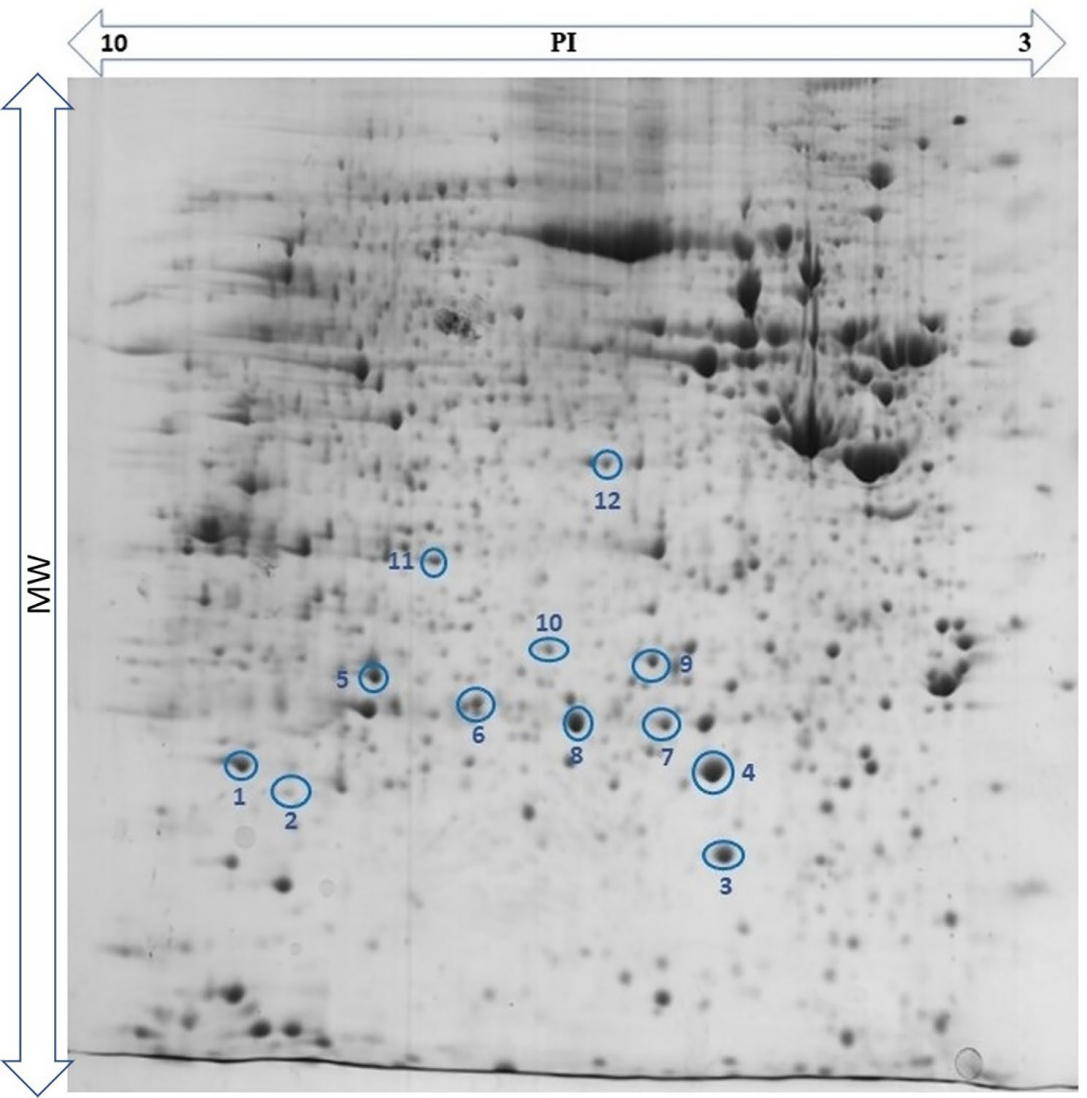
2-DE gel image. The differentially expressed protein spots are numbered as same in Table 2

Gel images were grouped in NMIBC, MIBC, and adjacent non-cancerous tissues. The protein gel map of the NMIBC group was considered as a reference for protein matching and comparing other groups. The results of tissue proteome analysis revealed 12 protein spots with significant differential expression among NMIBC, MIBC, and adjacent non-cancerous groups. These spots of interest were located at approximately PI 5 to 9 and MW 21 to 38 kDa (Fig. 1). Nine spots were up-regulated in NMIBC and three of them were up-regulated in MIBC patients. The list of proteins identified by Uniprot databases and tandem MS analysis data set are shown in Supplementary Table 1. Protein lists with identifications > 1 per spot were ranked by protein sequence coverage, number of identified peptides per protein, and the theoretical MW-pI based on the associated human accession number.

**Table 2:** Protein spots identified by Uniprot databases and MS analysis data set of the bladder cancer tissue samples

No	Protein name	Spot No.	PI	Theoretical MW [kDa]
NMIBC				
1	Peroxiredoxin (PRDX1)	1	8.27	22,11
2	Pyrimidine nucleoside monophosphate kinase (UMP/CMPK)	3	5.44	22,22
3	Glutathione S-transferase M1 (GSTM1)	4	5.43	23,356
4	Phosphoglycerate mutase 1 (PGAM1)	5	6.67	28,804
5	Peroxiredoxin (PRDX6)	8	6	25,035
6	Proteasome activator complex subunit1 (PSME1)	9	5.78	28,723
7	Heat shock protein beta-1 (HSPB-1)	10	5.98	22,782
8	Annexin-I (ANXA1)	11	6.57	38,714
9	Macrophage-capping protein (CAPG)	12	5.82	38,498
**MIBC**				
10	Biliverdin Reductase B (Flavin Reductase) (BLVRB)	2	7.13	22,119
11	Peroxiredoxin (PRDX2)	6	5.66	2,892
12	15-hydroxyprostaglandin dehydrogenase (HPGD)	7	5.56	28,977

pI – isoelectric point; MW, molecular weight; non-muscle-invasive bladder cancer, NMIBC; muscle-invasive bladder cancer, MIBC.

### Differential protein expression profiles

Protein spots identified by LC-MS were biliverdin reductase B (BLVRB), NAD+-dependent 15-hydroxyprostaglandin dehydrogenase (PGDH or HPGD), 3 molecular isoforms of peroxiredoxin (PRDX1, 2, and 6), glutathione s-transferase M1 (GSTM1), proteasome activator complex subunit 1 (PSME1), heat shock protein beta-1 (HSPB-1), macrophage-capping protein (CAPG), pyrimidine nucleoside monophosphate kinase (UMP/CMPK), annexin-I (ANXA1), phosphoglycerate mutase 1 (PGAM1). Twelve selected spots were identified as 9 proteins and 3 isoforms proteins. Twelve identified proteins are shown in Table 2.

**Fig. 2:**
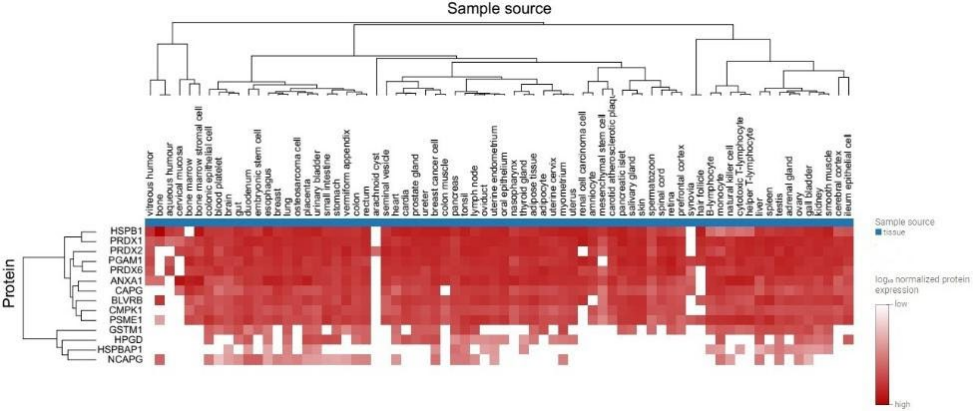
Expression heatmap. Compare of 12 identified proteins expression over various tissues

We have also examined the ProteomicsDB database (https://www.proteomicsdb.org) with the list of our 12 identified proteins for insight that these proteins had been detected in which tissues. The results of our analysis showed, these proteins are expressed in most other tissues. According to this map, the HSPB1 protein has a high expression level and ten proteins (BLVRB, PRDX1, PRDX2, PRDX6, GSTM1, PSME1, CAPG, CMPK1, ANXA1, PGAM1, and PGDH) have low to medium expression levels in normal bladder tissue (Fig. 2, the eighteenth column).

### Biological, functional, and pathway analysis

**Fig. 3:**
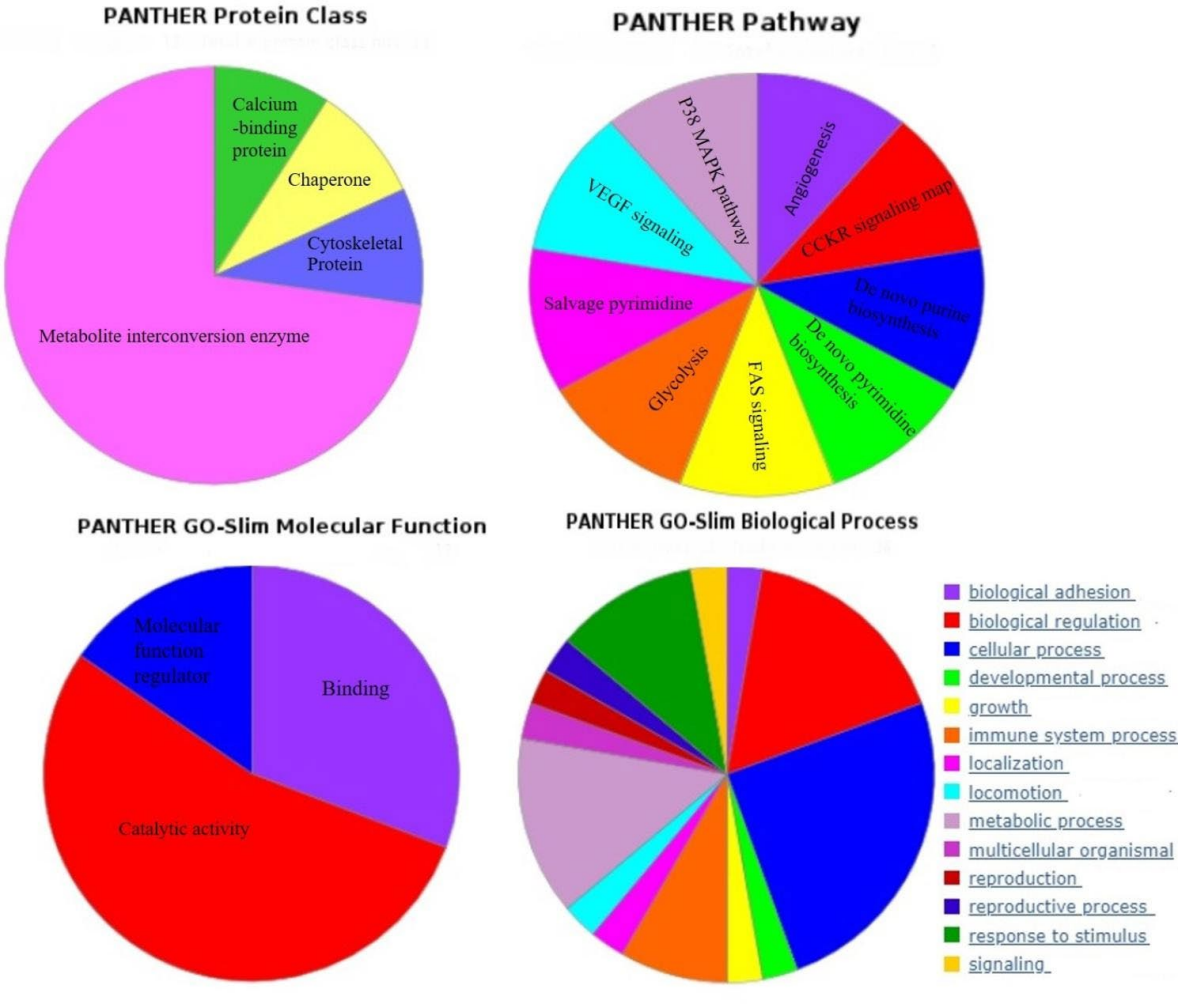
PANTHER classification of twelve identified proteins

To gain insight into the biological functions associated with identified proteins in GO categories PANTHER classification was performed (FDR P < 0.05). As a result of this analysis, the detected proteins were found to be involved in cell activation, cell redox homeostasis, cellular response to chemical stimulus, cellular detoxification, response to reactive oxygen species (ROS), and response to oxidative stress. Also, GO cellular component term analysis revealed these proteins are significantly expressed in the extracellular exosome/region and cytosol compartments. These proteins generally functioned as peroxiredoxin, oxidoreductase, peroxidase, thioredoxin peroxidase, and cadherin binding activity (P < 0.05) (Fig. 3). The list of GO enriched in proteins is summarized in Supplementary Table S2.

### Protein-Protein Interaction Network Analyses

**Fig. 4:**
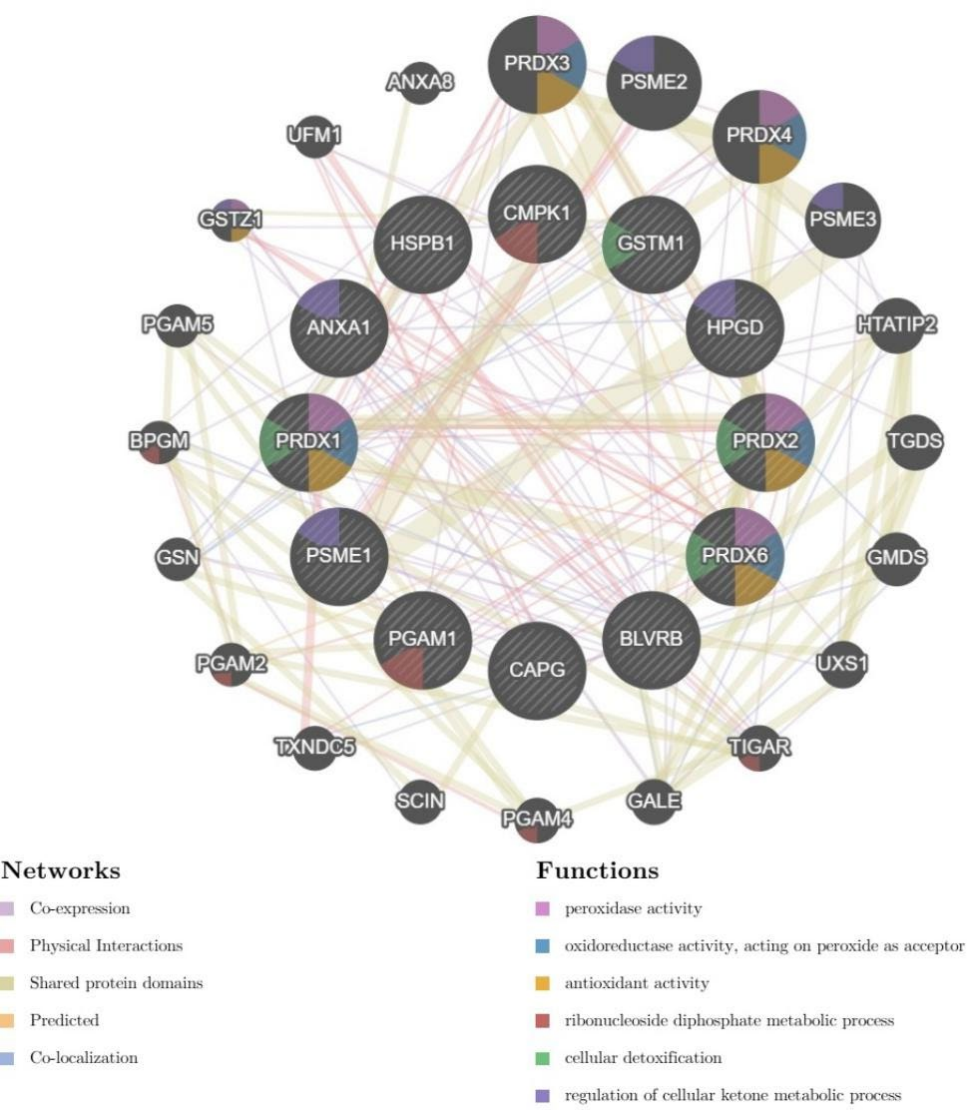
Protein-protein interaction and functional characteristics between identified proteins and the other proteins constructed by GeneMANIA. Nodes with a gray diagonal are identified portions; black nodes are related proteins; Colors in nodes is related to a function

**Fig. 5:**
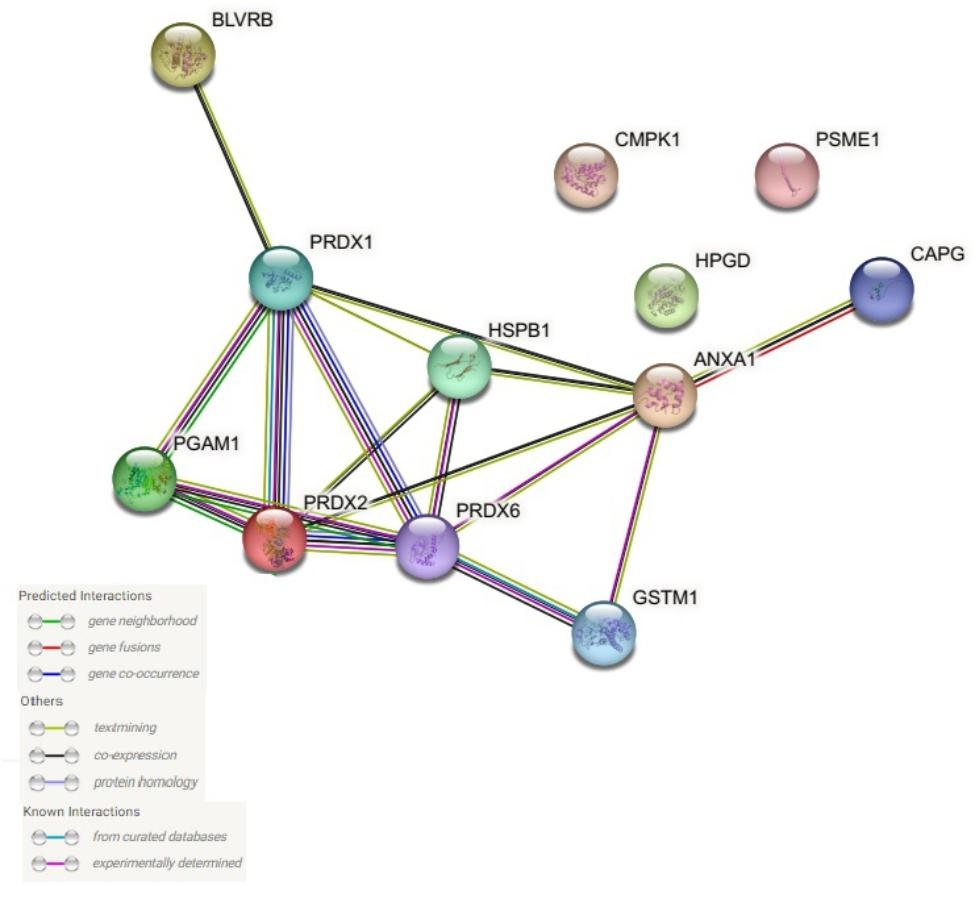
STRING diagram. Protein–protein interaction (PPI) network derived from the 12 proteins related to UBC tissue. Colored nodes are query proteins and first shell of interactors

The possible relationship between the identified proteins was analyzed using the GeneMANIA database. According to the GeneMANIA database, the highest interaction weight in our analysis was related to the co-expression of ANXA-1 and BLVRB (p = 0.001) as well as physical interactions between PRDX6 and HSPB1 (p = 0.002). The most significant functions were associated with the peroxidase activity (FDR = 1.02e-7), oxidoreductase activity (FDR = 1.13e-6), antioxidant activity (FDR = 4.23e-6), nucleoside diphosphate metabolic process (FDR = 7.92e-5), cellular detoxification (FDR = 3.60e-4), and regulation of cellular ketone metabolic process (FDR = 3.60e-4). Furthermore, the network showed co-expression link weight between BLVRB and ANXA1 (p = 0.001) (Fig. 4). Also, STRING database was used to study a possible relationship between the differentially expressed proteins and PPI with a high local clustering coefficient (< 0.7). The network created an interaction between the total 12 nodes and 17 edges with an average node degree of 2.83 and a PPI enrichment (P < 1.0e-16) (Fig. 5). These clusters share interactions among themselves, indicating that these molecules could play key roles in diverse pathways that contribute to UBC pathology. In addition, the detoxification of ROS and cellular responses to stress were the most significant Reactome pathways among identified proteins (FDR = 1.76e-05 and 0.0124, respectively). All Reactome topology pathways obtained from over-representation analysis are shown in Supplementary Table S3. These pathways are ranked according to the p-value and FDR.

## Discussion

In the current study, we performed a comparative 2-DE based proteomics analysis combined with LC-MS/MS protein identification in UBC proteins obtained from NMIBC and MIBC tissue specimens to identify candidate protein biomarkers to be used as non-invasive diagnostic and prognostic tools. We employed an integrated analysis to identify biomarkers that may be associated with disease progression and prognosis. Our results revealed that despite the overall similarity in protein expression pattern, at least twelve proteins were differentially, and reproducibility expressed. Nine proteins up-regulated in NMIBC tissues (PRDX1, PRDX6, GSTM1, PSME1, CAPG, CMPK1, ANXA1, PGAM1, and HSPB1), and three proteins up-regulated in MIBC (PRDX2, BLVRB, and HPGDH). UBC is a highly heterogeneous malignancy at the cellular, genomic, transcriptional, and molecular alterations levels which lead to differences in phenotypical characteristics and resistance to therapies [21]. Our data demonstrated some differentially expressed proteins which may improve early diagnosis of UBC patients.

According to the pathway analysis obtained from the current study, a vast range of aberrantly expressed proteins are related to the processes of cellular response to stimuli such as chemical stress and reactive oxygen species. Overexpression of these proteins in cancerous bladder tissue may reflect their protective role in detoxification process of chemical compounds e.g., what is found in tobacco smoke [22]. On the other hand, immunohistochemistry (IHC) validation analysis by the Human Protein Atlas (PHA) shows that CAPG has not been previously reported in UBC tissue specimens, the rest of identified proteins in this study had been shown to have low to high expression levels in UBC tissue.

Previously, it has been found that dysregulation of the cellular redox homeostasis in pursuit of increasing levels of ROS leads to molecular damage that promotes various pathological processes of tumorigenesis [23]. Notably, most of the identified proteins in this study were involved in oxidative stress. PRDXs are antioxidant enzymes which involved in several biological processes cell growth, immune response, as well as intracellular redox homeostasis, and antioxidant function [24]. PRDX1 is a major member of the antioxidant enzymes involved in many cellular signaling pathways. Anti-apoptotic activity of PRDX1 reduces the intracellular accumulation of ROS that plays a pivotal role in the progression and invasion of various cancers [25, 26]. The results of some studies have previously demonstrated that antioxidant systems are activated to combat oxidative stress induced by ROS in cancer cells [25, 27].

In this study, we found expression of PRDX1 was significantly elevated in NMIBC compared to MIBC tissues. In line with our results, the positive expression levels of the PRDX1 in bladder tumor specimens and its association with recurrence and survival rates of UBC patients had been reported [28]. In a proteomics study, overexpression of PRDX1 was significantly associated with poor prognosis in ovarian cancer [29].

We also observed a significant increase in the expression of PRDX6 in NMIBC patients. Previous studies showed elevated expression levels of PRDX6 in a variety of metastatic potential, and poor prognosis cancers [30, 31]. Additionally, there is direct evidence that PRDX6 modulates the JAK-STAT3 pathway which enhances the proliferation of bladder tumor cells [27]. In another study, overexpression of PRDX6 promoted pathological tumorigenesis conditions in cervical cancer cells [30].

In parallel, PRDX2 is the most efficient endogenous antioxidant compared with the other antioxidants which has both tumor suppressor and tumor promoting activities [32]. Moreover, the effects of PRDX2 regulation on intracellular signaling of PRDX6 have been correlated with different aspects of tumor behavior such as invasive, apoptosis, as well as, resistance to chemotherapeutic agents and tumor recurrence [33, 34]. The oncogenic property of PRDX2 by degradation of p53 has previously been suggest [35]. On the contrary, PRDX2 acts as a tumor suppressor molecule depending on the tumor type and tumor stage [32]. Also, a comparative urine proteomics study reported the evaluation of PRDX2 in the urine sample of bladder cancer patients in different stages which confirms our study [36].

In this study, we found that expression of PRDX1 and PRDX6 was significantly elevated in NMIBC compared to MIBC tissues. Whereas, PRDX2 was significantly increased in MIBC tissues. It seems that the PRDX1 and PRDX6 may be considered diagnostic and prognostic biomarker for UBC and up-regulation of these proteins can be correlated with unfavorable prognosis in UBC patients. In similar, high PRDX2 expression in MIBC patients had a worse prognosis. However, the biological functions of these three PRDXs in UBC have not been completely explored.

We also found PGAM1 which is a glycolytic enzyme stimulates anabolic pathways and keeps redox balance under hypoxic conditions. Increasing evidence supports that aberrant expression of PGAM1 in several cancers promotes tumor growth and progression [37–39]. PGAM1 regulates Hypoxia-inducible factor 1α (HIF-1α) and PI3K/Akt/mTOR pathways that in turn can provide required energy for proliferation, invasion, and metastasis of tumor cells [40]. In a 2-DE and ESI-Q-TOF MS/MS-based proteomic study PGAM1 significantly up-regulated in UBC tissue compared with adjacent normal tissue [41]. It has been shown that PGAM1 silencing can suppress the oncogenic effect of mammalian target of rapamycin (mTOR)-mediated tumorigenesis [42].

In parallel, the BLVRB is a multifunctional enzyme that mediated redox regulation and is known to regulate the activity of kinase signaling pathways in a diverse manner [43]. Moreover, BLVRB is recognized as a major endogenous antioxidant enzyme which predominantly expresses in the embryonic stage. Interestingly, it was reported that overexpression of BLVRB indicates the early stage of tumor development which highlights the role of hypoxia condition in the tumor induction [44]. However, there has been limited evidence on the association of BLVRB proteins and their possible pro-carcinogenic mechanism in cancers. Overexpression of BLVRA protein had been detected in prostate cancer tissue as a diagnostic biomarker by MALDI-MS [45]. Significantly, the oncogenic function of BLVRB has not been evaluated in UBC and our study is the first report of BLVRB up-regulation in UBC by proteomics method.

High expression levels of GSTP1 have been reported in relation to tumor growth, drug resistance, and carcinogenesis in colorectal [46], prostate [47], and breast cancer [48]. In line our data, the up-regulation of GSTP1 contributes to an increase in detoxification functions and altered apoptotic pathways of urothelial bladder cells [49]. It is interesting that exposure to potentially carcinogenic compounds such as benzo[a]pyrene and tobacco smoke induces the expression of GSTP1 to eliminate toxic substances in urothelial bladder cells [50, 51]. However, GSTP1 not only has both physiologic and pathologic functions but also can be considered as a potential marker for UBC. PSME1 was another protein that we identified in associated with cell detoxification. PSME1 is involved in virtually all cellular processes and participates in many essential cellular processes like cell cycle, protein quality control, immune responses, as well as cell survival or death by its proteostasis function [52]. Accordingly, increasing evidence has shown that up-regulated PSME1 has been considered as a prognostic parameter and also a factor involved in the tumorigenesis [53]. Affinity chromatography followed by MS analysis study reported the high expression of PSME1 correlated with the primary and metastatic phases in prostate cancer [54]. Similarly, high expression levels of PSME1 were associated with favorable overall survival of skin cutaneous melanoma [55]. Although, the exact role of PSME1 in the tumor progression and its possible role in UBC immunotherapy has not yet been assessed.

Additionally, HSPB1 is recognized as a chaperone expressed in response to different kinds of cellular stress [56]. Besides, changes in HSPB1 expression are linked to tumor growth, metastatic potential, deleterious resistance to chemotherapy, tumor aggressiveness, and poor clinical outcomes in various types of carcinomas [57, 58]. This protein with antiapoptotic activity and tumorigenic properties is considered an important therapeutic target in cancer [59]. HSPE1 was significantly elevated in urine specimens of patients with UBC and regarded as a potential protein marker for early detection [60]. Nevertheless, in a conflicting result, HSBP1 knockdown revealed a significant association with adverse pathological characteristics of UBC cell lines although the authors have not observed any association with apoptosis effect, chemotherapeutic sensitivity, and clinical outcomes on tumor cells [61]. In the present study, we observed up-regulation of HSPB1 in NMIBC patients therefore, our date supports the previous notion that this biomarker may be a useful tool for early detection of UBC.

Increasing cell motility by up-regulation of CAPG might also lead to tumorigenesis and enhanced metastasis in bladder transitional cell carcinoma [62]. CAPG knockdown significantly inhibited cell cycle, migration, and progression through CDC42 and the extracellular signal-regulated kinase 1/2 ERK/ the mitogen-activated protein kinase (ERK/MAPK) signaling [41]. In line with our study, the positive expression of CAPG protein identified by 2-DE and LC-MS/MS analysis was associated with pathophysiological characteristics and a high recurrence rate in MIBC patients [63]. Therefore, high CAPG expression could be related to clinical stage and poor prognosis.

Furthermore, studies have described that localization of the CMPK1 (UMP/CMPK) in the nucleus and cytoplasm could accrue in several cancer cells which have also been identified as a prognostic marker [64, 65]. A previous study identified and validated CMPK1 as a prognostic marker for triple-negative breast cancer by MS [66]. CMPK1 was overexpressed in human epithelial-type ovarian cancer through the TGF-β signaling pathway [67]. These results suggest that CMPK1 could serve as a prognostic biomarker in bladder cancer.

ANXA1 by activating the EGFR signaling cascade facilitates the proliferation, invasion, and migration of UBC cells that ultimately led to the progression and poor prognosis [68]. Studies in bladder tumor tissues have shown that ANXA1 had prognostic value in MIBC patients [69]. In a recent study by LC-MS shotgun analysis, ANXA1 was introduced as a predictor in patients with different relapse risks [70]. Up-regulation of ANXA1 was also reported with a role in a variety of tumor development processes and metastasis-free survival in high-grade compared to low-grade UBC [71].

Finally, we identified the up-regulation of PGDH in MIBC tissues. As demonstrated in several studies, the expression of the PGDH has been significantly decreased in various types of cancer tissues which assuming to act as a potential tumor suppressor by inhibiting proliferation and differentiation of cancer cells [72–74]. While most reports revealed low expression levels of PGDH in different cancers, high expression levels of PGDH have been observed in ovarian cancer [75]. Also, high expression of PGDH promoted invasion in breast cancer [76]. Nevertheless, there are limited studies on PGDH expression in urinary bladder cancer. The PGDH expression inhibition contributes to increased malignancy in the well-differentiated bladder cancer cell line [77]. Nevertheless, there are limited studies on PGDH expression in UBC. Furthermore, the biological role and the function of PGDH as a tumor suppressor have not yet been explored in UBC.

Our study identified several target proteins that show promise as potential biomarkers for UBC. The identification of these proteins as potential biomarkers has important implications for clinical practice, as they may be used for earlier detection, prognosis, and monitoring of UBC. Further comparative analyses within the UBC patient population, particularly among different stages or grades are needed to confirm these findings.

## Conclusion

Taken together, our study represents UBC comparative proteome analysis in NMIBC and MIBC. Our data confirms the results of previous studies, however most of those were limited to western blot analysis, gene expression, and IHC methods. Collectively, contradictions are observed in the results of previous studies, which can be due to the differences in the pathological conditions of each tumor, differences among methods, and the contribution of different genetic backgrounds of studied populations.

The small sample size in the MIBC group was one of the limitations of this study which largely enrolled by strict criteria of not using any therapeutic intervention and identifying the patients with the first sign of hematuria. Moreover, the use of adjacent non-cancerous tissue may not be desirable due to limited access to normal bladder samples owning to genetic abnormalities. Based on the National Cancer Institute’s Early Detection Research Network (NIH), our identified proteins have not been investigated for routine clinical practice yet. Hence, the validation of identified proteins, particularly using studies with a very large clinical cohort to evaluate their potential clinical utilities in diagnosis or prognosis, could be useful for further verification and quality control.

## Electronic supplementary material

Below is the link to the electronic supplementary material.

Supplementary Table S1: The list of proteins identified by Uniprot databases and tandem MS analysis data set

Supplementary Table S2: The list of GO enriched in 12 identified proteins

Supplementary Table S3: Reactome topology pathways obtained from over-representation analysis

## Data Availability

The data that support the findings of this study are available on request from the corresponding author. All data generated or analyzed during this study are included in this published article.

## Abbreviations

NMIBC: Non-muscle-invasive bladder cancer
MIBC: Muscle-invasive bladder cancer
UBC: Urothelial bladder cancer
2-DE: Two-dimensional electrophoresis
MS: Mass spectrometry
FDR: False discovery rate
TURB: Transurethral resection of bladder tumor
LC-MS: Liquid chromatography–mass spectrometry
UICC: Union for International Cancer Control
DTT: Dithiothreitol
CHAPS: 3-[(3-cholamidopropyl) dimethylammonio]-1-propanesulfonate
IPG: Immobilized pH gradient
BSA: Bovine serum albumin
IEF: Isoelectric Focusing
PI: Isoelectric point
MW: Molecular weight
CBB: Coomassie brilliant blue G-250
PPI: Protein-protein interaction
BLVRB: Biliverdin reductase B
PGDH: NAD+-dependent 15-hydroxyprostaglandin dehydrogenase
PRDX: Peroxiredoxin
GSTM1: Glutathione s-transferase M1
PSME1: Proteasome activator complex subunit 1
HSPB-1: Heat shock protein beta-1
CAPG: Macrophage-capping protein
UMP/CMPK: Pyrimidine nucleoside monophosphate kinase
ANXA1: Annexin-I
PGAM1: Phosphoglycerate mutase 1
ROS: Response to reactive oxygen species
IHC: Immunohistochemistry
PHA: Human Protein Atlas
HIF-1α: Hypoxia-inducible factor 1α
mTOR: Mammalian target of rapamycin
ERK: Extracellular signal-regulated kinase
MAPK: Mitogen-activated protein kinase
GO: Geneontology
NIH: National Cancer Institute’s Early Detection Research Network
