# Supplementary material for: Comparative proteomics analysis in different stages of urothelial bladder cancer for identification of potential biomarkers: highlighted role for antioxidant activity
Source: Clin Proteomics. 2023 Jul 27;20:28. doi: 10.1186/s12014-023-09419-8 (PMC10373361; doi:10.1186/s12014-023-09419-8)
Supplement: Supplementary file 1 — Supplementary Table S1: The list of proteins identified by Uniprot databases and tandem MS analysis data set [file 12014_2023_9419_MOESM1_ESM.docx]

**Table S1.** Up-regulated protein spots identified in NMIBC and MIBC bladder cancer tissue samples.

| **Spot**  **No.** | **Protein name** | **UniProt** IDs | **Gene name** | **Sequence coverage (%)** | **Unique peptides** | **PI** | **Theoretical**  **MW [kDa]** |
| --- | --- | --- | --- | --- | --- | --- | --- |
| 1 | Peroxiredoxin (PRDX1) | [Q06830](https://www.uniprot.org/uniprotkb/Q06830/entry) | PRDX1 | 95,5 | 25 | 8.27 | 22,11 |
| 2 | Biliverdin Reductase B (Flavin Reductase) (BLVRB) | [P30043](https://www.uniprot.org/uniprotkb/P30043/entry) | BLVRB | 84.5 | 16 | 7.13 | 22,119 |
| 3 | Pyrimidine nucleoside monophosphate kinase (UMP/CMPK) | [P30085](https://www.uniprot.org/uniprotkb/P30085/entry) | CMPK1 | 70 | 15 | 5.44 | 22,22 |
| 4 | Glutathione S-transferase M1 (GSTM1) | [P09488](https://www.uniprot.org/uniprotkb/P09488/entry) | GSTM1 | 71,9 | 15 | 5.43 | 23,356 |
| 5 | Phosphoglycerate mutase 1 (PGAM1) | [P18669](https://www.uniprot.org/uniprotkb/P18669/entry) | PGAM1 | 81 | 23 | 6.67 | 28,804 |
| 6 | Peroxiredoxin (PRDX2) | [P32119](https://www.uniprot.org/uniprotkb/P32119/entry) | PRDX2 | 90.4 | 24 | 5.66 | 2,892 |
| 7 | 15-hydroxyprostaglandin dehydrogenase (HPGD) | [P15428](https://www.uniprot.org/uniprotkb/P15428/entry) | HPGD | 84.2 | 24 | 5.56 | 28,977 |
| 8 | Peroxiredoxin (PRDX6) | [P30041](https://www.uniprot.org/uniprotkb/P30041/entry) | PRDX6 | 82,6 | 29 | 6 | 25,035 |
| 9 | Proteasome activator complex subunit1 (PSME1) | [Q06323](https://www.uniprot.org/uniprotkb/Q06323/entry) | PSME1 | 82.3 | 22 | 5.78 | 28,723 |
| 10 | Heat shock protein beta-1 (HSPB-1) | [P04792](https://www.uniprot.org/uniprotkb/P04792/entry) | HSPB1 | 93.7 | 28 | 5.98 | 22,782 |
| 11 | Annexin-I (ANXA1) | [P04083](https://www.uniprot.org/uniprotkb/P04083/entry) | ANXA1 | 79 | 32 | 6.57 | 38,714 |
| 12 | Macrophage-capping protein (CAPG) | [P40121](https://www.uniprot.org/uniprotkb/P40121/entry) | CAPG | 74 | 20 | 5.82 | 38,498 |

pI – isoelectric point; MW, molecular weight. Protein score of all proteins is 323.31.
